# Identification of Key Residues in the NisK Sensor Region for Nisin Biosynthesis Regulation

**DOI:** 10.3389/fmicb.2017.00106

**Published:** 2017-01-26

**Authors:** Xiaoxuan Ge, Kunling Teng, Jian Wang, Fangyuan Zhao, Jie Zhang, Jin Zhong

**Affiliations:** ^1^State Key Laboratory of Microbial Resources, Institute of Microbiology, Chinese Academy of SciencesBeijing, China; ^2^University of Chinese Academy of SciencesBeijing, China

**Keywords:** histidine kinase, NisK, peptide pheromone, lantibiotics, nisin

## Abstract

Histidine kinase (HK) NisK is well known to sense lantibiotic nisin for regulating the biosynthesis of nisin. NisK possesses two trans-membrane segments and a large extracellular region and nisin contains 34 amino acids with five lanthionine rings. Unlike most peptide sensing HK with multi trans-membrane segments, NisK is a representative of a group of rarely reported HK that sense peptide as ligand. To reveal how NisK senses nisin molecule to regulate nisin biosynthesis, we constructed a reporter *Lactococcus lactis* strain with *nisRK* constitutively expressed and a reporter gene *lacZ* expressed under the control of promoter P*_nisA_*. We showed that the extracellular region of NisK was involved in recognizing nisin. Conserved residues in this group of HK were found in the extracellular region of NisK and mutagenesis of these residues in the reporter strain revealed that several hydrophobic residues including two aromatic residues are crucial for NisK sensing nisin and regulating nisin biosynthesis. Substitutions of hydrophobic regions in NisK extracellular domain showed that the first strand that was rich of hydrophobic amino acids was involved in regulating nisin biosynthesis. A negatively charged residue in the first βstrand also contributed to nisin biosynthesis. Protein binding analyses demonstrated that nisin could not interact with key NisK mutants, indicating these site in the extracellular region of NisK was involved in recognizing nisin.

## Introduction

Nisin is the most representative lantibiotic produced by *Lactococcus lactis* and is widely used as a natural and safe food preservative in many countries (Gharsallaoui et al., 2015a,b). Nisin is also potentially used for therapeutic purposes, such as the treatment of clinical bovine mastitis, inhibition of head and neck cancer tumorigenesis, and so on (Cao et al., 2007; Wu J. et al., 2007; Field et al., 2015; Kamarajan et al., 2015). The gene cluster related to nisin biosynthesis includes 11 genes *nisABTCIPRKFEG*. *nisA* encodes nisin precursor in which serine and threonine residues are dehydrated to dehydroalanine and dehydrobutyrine by NisB and cyclized with cysteines by NisC to form thioether bridges. Nisin precursors are exported across the cell membrane by NisT and the leader peptide could be cleaved by protease NisP, releasing mature nisin. Nisin belongs to the group of AI lantibiotics and its structure has been elucidated as a linear peptide containing five intermolecular lanthionine rings (Chatterjee et al., 2005). Nisin is the first found bifunctional lantibiotic which could not only work as antimicrobial agent, but also act as signal to induce its own biosynthesis through the quorum sensing system NisK/R two-component regulatory system (Kuipers et al., 1995).

Two-component system (TCS) is the most prevalent regulation system in bacteria and exists in almost all bacteria, some lower eukaryotes and plants (Wuichet et al., 2010; Schaller et al., 2011). Microbes utilize TCS to sense environmental signals and quickly regulate necessary gene expression to survive in various niches. Typical TCS compose of a histidine kinase (HK) and a cognate response regulator (RR) (Stock et al., 2000). The sensor C can sense specific stimuli and then be auto-phosphorylated on a conservative histidine residue. The activated HK transfers the phosphate group to its cognate RR on the conservative aspartic acid residue followed by the initiation of the downstream genes expression (Stock et al., 2000). The C-termini of HKs include two typical conservative domains, the dimerization and histidine phosphotransfer (DHp) domain, which contains the conserved histidine involved in auto-phosphorylation, and the catalytic and ATP binding (CA) domain. The N-terminals of HKs are usually their sensor regions which vary greatly from their sequences, structures and membrane topologies to identify various environmental stimuli specifically. The HK are classified into three major groups according to the architectures of their sensor regions. The first group members possess one extracellular sensor domain flanked by two transmembrane segments. The second group HK usually have multi-transmembrane segments and their sensing regions are associated with their transmembrane helices. The third group HK contain cytoplasmic sensor domains (Mascher et al., 2006).

Up to date, 1000s of TCS components have been revealed, whereas most studies of HK are limited to their conserved cytoplasmic regions because of the difficulty of the expression and structural analysis of membrane proteins. The known extracellular sensor domains of Group I HK contain mixed alpha-beta folds (represented by PDC domain), all alpha folds (represented by NarX sensor domain), and sensor domains that show a similar fold to extracellular binding proteins (represented by HK29s) (Cheung and Hendrickson, 2010), and most of these HK are in Gram-negative bacteria and their corresponding ligands are often ions or small molecules (Reinelt et al., 2003; Cheung and Hendrickson, 2009). The Gram-positive bacteria usually recognize peptides as quorum sensing pheromones to activate TCS or other regulatory cascades to control its own biosynthesis or other cellular processes (Mascher et al., 2006). Most peptide sensing HK, like auto-inducing peptide (AIP) receptor AgrC and class AII lantibiotic bovicin HJ50 receptor BovK, were found to have multi-transmembrane segments, and their sensing regions were associated with the cell membrane (Jensen et al., 2008; Teng et al., 2014; Wang et al., 2014). There is few report about the mechanism of Group I HK involving in sensing peptide ligand.

Nisin is a 34-amino acid lantibiotic peptide with several unusual dehydro residues and five lanthionine ring structures (Chatterjee et al., 2005; Zhang et al., 2012). Our previous work has reported that the N-terminal ring structures (ring A and ring B) in nisin were involved in activating NisK to act as an inducing molecule (Ge et al., 2016). In this study, we described the sensing characteristics of NisK which is representative of a rarely reported group of HK. These proteins possess large extracellular sensor regions and usually sense lantibiotic peptide pheromones as ligands. we revealed that the extracellular region of NisK was involved in sensing nisin and some conservative hydrophobic sites and one negative charged residue in NisK extracellular sensor region were necessary to recognize nisin.

## Materials and Methods

### Bacterial Strains and Growth Conditions

Strains and plasmids related to this study are listed in **Table 1**. *L. lactis* CV56 (Gao et al., 2011), MG1363 (Gasson, 1983), as well as their derivatives were routinely cultured in GM17 medium (M17 medium with 0.5% (w/v) glucose added) at 30°C with 5 μg/ml erythromycin added. *Escherichia coli* DH5α, BL21 (DE3), and MC1061 (Casadaban and Cohen, 1980) were maintained in Luria-Bertani (LB) broth at 37°C with 10 μg/ml chloramphenicol, 100 μg/ml erythromycin or 100 μg/ml ampicillin added if necessary. *E. coli* DH5α and MC1061 were used as hosts for transformation of pMG36e (van de Guchte et al., 1989), and *E. coli* BL21 (DE3) was used to express the cytoplasmic portion of NisK protein.

**Table 1:** Bacterial strains and plasmids used in this study.

Strain or plasmid	Relevant characteristics	Reference
**Strains**		
*Lactococcus lactis* CV56	Nisin A production strain	Gao et al., 2011
*Lactococcus lactis* MG1363	Plasmid free; no nisin production related genes	Gasson, 1983
*Lactococcus lactis* MG1363 RKNlacZ	*Lactococcus lactis* MG1363 with pMG36e-RKNlacZ	This work
*Lactococcus lactis* MG1363 RKNlacZ-ΔKe	*Lactococcus lactis* MG1363 with pMG36e-RKNlacZ-ΔKe	This work
*Escherichia coli* BL21 (DE3)	Protein purification strain	Promega
*Escherichia coli* MC1061	Cloning host	Casadaban and Cohen, 1980
**Plasmids**		
pMG36e	*E. coli*–*L. lactis* shuttle vector, Em^r^	van de Guchte et al., 1989
pMG36e-RKNlacZ	pMG36e with *nisRK* and P*_nisA_-lacZ* inserted	This work
pMG36e-RKNlacZ-ΔKe	pMG36e-RKNlacZ with residues from Ile39 to Ser140 deleted	This work
pMG36e-NlacZ	pMG36e P*_nisA_-lacZ*	This work

### Plasmid Constructions

*nisRK* and P*_nisA_* were amplified from the genome of *L. lactis* CV56, the P*nisA-lacZ* DNA fragments were obtained by fusion PCR. Plasmids, PCR products, or DNA fragments were purified by Axygen kits (Axygen, USA). *nisRK* was amplified with primers containing recognition sites of *Pst*I (PRF: tgcaCTGCAGtctgtacgaagtcaagaaattttacctg) and *EcoR*I (PKR: cgGAATTCatatgttttagaaagattttgaatttttac) and P*_nisA_-lacZ* was amplified and fused with primers containing recognition sites of *Pst*I and *Hind*III. The primers used for P*_nisA_-lacZ* fragment are PnisF (cccAAGCTTatataggtttattgagtcttagacatac), PnisR (cgggatccattttgagtgcctccttataatttattttg), PlacF (ggcactcaaaatggatcccgtcgttttacaacgtcgtg), and PlacR (tgcaCTGCAGttatttttgacaccagaccaactgg). The two segments *nisRK* and P*nisA-lacZ* were inserted into plasmid pMG36e getting pMG36e-RKNlacZ. When only segment P*_nisA_-lacZ* was inserted into pMG36e, we got plasmid pMG36e-NlacZ. Site-directed mutagenesis of *nisK* were performed according to the protocols reported (Chiu et al., 2004), generating different pMG36e-RKNlacZ derivatives. Briefly, an inverse PCR amplification of the template was carried out by two tailed long primers and two short primers in a single reaction. The tailed primers were designed to contain the desired mutation on complementary overhangs at the terminus of PCR products. After post-amplification denaturation and re-annealing, heteroduplex formation between the mixed PCR products created the desired clonable mutated plasmid. All the PCR procedures were carried out using Phusion DNA polymerase purchased from Thermo Fisher. The restriction enzymes used in plasmid constructions were purchased from Thermo Fisher.

### LanKs Modular Architecture Research

Histidine kinases that are involved in lantibiotics biosynthesis are named LanKs. The structures of known LanKs which sense specific lantibiotics were analyzed by SMART database^1^ (Simple Modular Architecture Research Tool) (Letunic et al., 2006). Thirteen LanKs were predicted to have two *trans*-membrane segments and an extracellular region (>100 amino acids). **Table 2** showed their protein names, GenBank accession numbers, producing strains and the corresponding lantibiotics.

**Table 2:** Producing strains and corresponding lantibiotics of LanKs whose schematic structures are similar to NisK.

Histidine kinases (HK)	Accession number	Strains	Lantibiotics
NisK	YP_005867990.1	*Lactococcus lactis* CV56	Nisin
SpaK	AAB91593.1	*Bacillus subtilis* ATCC 6633	Subtilin
EtnK	AEK64500.1	*Bacillus subtilis* DSM 15029T	Entianin
SlvK	AEX55158.1	*Streptococcus salivarius* 5M6c	Salivaricin D
SrtK	BAB08161.1	*Streptococcus pyogenes* BL-T	Streptin 1/2
AAL15574.1	AAL15574.1	*Bacillus subtilis* A13	Ericin S/A
YP_001124399.1	YP_001124399.1	*Geobacillus thermodenitrificans*	Geobacillin I
McdK	CCF02675.1	*Streptococcus macedonicus* ACA-DC 198	Macedocin
MrsK	CAB60253.1	*Bacillus* sp. HIL-Y85	Mersacidin
ScnK	BAH87346.1	*Streptococcus mutans* NN2025	Mutacin K8
RumK	CAB93670.1	*Ruminococcus gnavus*	Ruminococcin A
SivK	ACX68641.1	*Streptococcus salivarius* 9	Salivaricin 9
ScnK	AAB92598.1	*Streptococcus pyogenes* FF22	Streptococcin A-FF22

### Assay of β-Galactosidase Activity

*Lactococcus lactis* MG1363 RKNlacZ and NisK mutated strains were incubated in GM17 overnight at 30°C and the cell cultures were transferred to a fresh GM17 medium with 1% inoculants. The cells were induced by nisin with a concentration of 9 ng/ml (2.68 nM) for 2 h when the OD_600_ of these strains reached 0.6–0.7. Induced cells of 2 ml were collected by two EP tubes with 1 ml per tube and washed once by Z buffer (0.06 M Na_2_HPO_4_⋅7H_2_O, 0.04 M NaH_2_PO_4_⋅H_2_O, 0.01 M KCl, 0.001 M MgSO_4_, 0.05 M β-mercaptoethanol, pH 7.0). One sample was resuspended in 1 ml Z buffer to measure OD_600_ while the other one was dissolved in 0.5 ml Z buffer with 10 mg/ml lysozyme at 37°C for 60 min to decompose the cell wall. Another 0.5 ml Z buffer with 0.1% SDS was added to the latter sample to dissolve cell membrane at 37°C for 15 min. The samples were centrifuged at 4°C with 1,200 rpm and the supernatant were collected for LacZ activity assay. Forty microliter ONPG was loaded into 200 μl supernatant or diluted supernatant to start reaction at 25°C. When the mixtures were yellow enough, 100 μl Na_2_CO_3_ (1M) was added to stop the reaction. The reaction time was recorded precisely. The values at OD_420_ and OD_550_ of each sample were measured to calculate the activity of the enzyme using the following equation: Miller Units = 1000 × [OD_420_–1.75 × OD_550_]/(T × V × OD_600_). OD_420_ and OD_550_ are read from the reaction mixture. OD_600_ reflects cell density in the washed cell suspension. T = time of the reaction in minutes. V = volume of culture used in the assay in mLs.

### Western Blotting Assay of NisK

The cytoplasmic portion of NisK (NisKc) was expressed in plasmid pET28a in *E. coli* BL21 (DE3), and His-tagged NisKc protein was purified by Ni^2+^-NTA affinity chromatograph column (GE healthcare). Purified NisKc was used as an antigen for antiserum production in mice (Institute of genetics, CAS). The cell lysates of *L. lactis* MG1363 RKNlacZ mutant was separated in SDS-PAGE gels and transferred to PVDF membrane. Western blotting analysis of NisK and NisK mutants were performed according to (Sambrook et al., 1989).

### Proteins Extraction

*Lactococcus lactis* MG1363 RKNlacZ mutant cells were harvested from 500 ml broth when OD_600_ reached 0.6–0.7, washed with PBS buffer (pH 7.4) once and resuspended in 20 ml PBS buffer. After lysing by sonication, cells were centrifuged at 6,000 *g* for 1 h to remove intact cells. The supernatant was ultra-centrifuged at 120,000 *g* for 2 h to separate soluble (cytoplasmic fraction) and insoluble (cell membrane fraction) proteins.

### Far-Western Blotting Assays

Nisin was labeled by biotin according to the method shown by (Koteva et al., 2010) in Beijing Biosynthesis Biotechnology Co., Ltd.. Briefly, nisin peptide was resolved by NaHCO_3_ (0.1 M, pH = 9.0) and incubated with biotin. Nisin labeled with biotin was purified by High Performance Liquid Chromatography and was named as b-nisin. The insoluble (cell membrane fraction) proteins were separated from *L. lactis* MG1363 RKNlacZ mutant cells. Far-Western blotting assays were performed using a modified protocol as described by (Wu Y. et al., 2007). Briefly, the insoluble proteins were firstly separated by SDS-PAGE, and transferred to a PVDF membrane as in a standard Western blotting. The proteins in the membrane were then denatured and renatured. The membrane is then blocked and probed with b-nisin. The horseradish peroxidase (HRP) conjugated streptavidin was used as the secondary antibody, and the lanes indicating b-nisin binding to NisK or NisK mutants were detected by photographic film.

### Surface Plasmon Resonance

The interaction between NisK extracellular region and nisin was checked by Surface Plasmon Resonance (SPR) conducted in BIACORE3000 (GE healthcare). The fragment of extracellular region of *nisK* (106–432) tagged by His_6_ was cloned into plasmid pET28a, and expressed in *E. coli* and purified. High concentration of signal peptide nisin was purified from commercial nisin product (Yinxiang, Zhejiang, China). His_6_-NisKex was dissolved in 10 mM acetate (pH 5.5) to a final concentration of 20 μg/ml, and immobilized on a CM5 chip by amide coupling to 1000 RU. Nisin dissolving in HBS-T buffer were injected through this chip as a analyte at a flow rate of 30 μl/min for 180 s followed by eluted with HBS-T buffer for 360 s. The concentrations of analyte were in twofold gradient dilution from 260 μM to 8.125 μM. After every detection, the chip was regenerated by injection of 50 mM NaOH at a flow rate of 10 μl/min for 100 s. All tests were performed at 25°C. Collected data were analyzed by BIAevaluation 4.1 software (GE Healthcare). The SPR curves were fitted by a 1:1 (Langmuir) binding model and local fitted *R*max to get the equilibrium constants *KD*, which was regarding to the protein–protein affinity.

## Results

### NisK Is Representative of a Rarely Reported Group of HK

**FIGURE 1:**
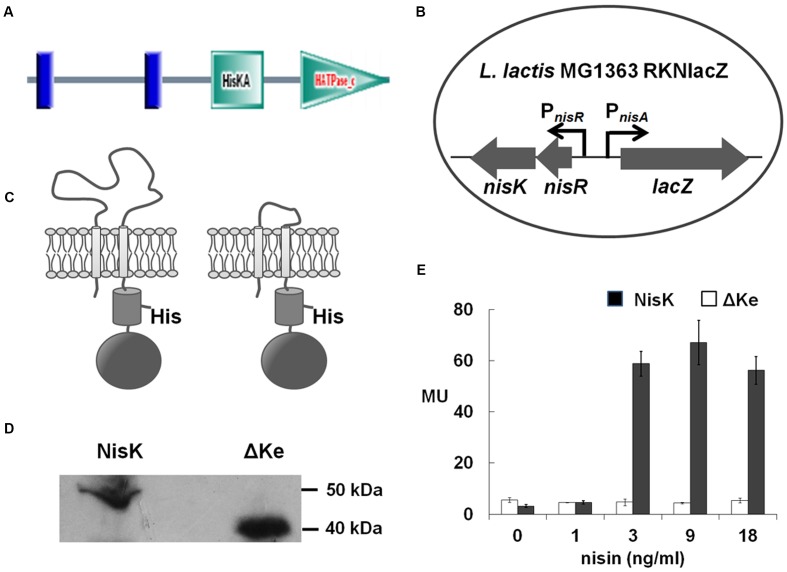
**NisK extracellular domain was involved in regulating nisin biosynthesis. (A)** Domain architectures of NisK predicted by SMART. Vertical lines indicate the predicted trans-membrane segments; HisKA indicates the His kinase phosphoacceptor domain that is a dimerization and phosphoacceptor domain of histidine kinase (HK); HATPase_c indicates the HK-like ATPase. **(B)** Schematic drawing of the reporter strain *Lactococcus lactis* MG1363 RKNlacZ for detecting nisin production regulated by NisK and NisK mutants. *lacZ* expression was controlled by *nisA* promoter (P*_nisA_*) and *nisRK* expression was controlled by *nisR* promoter (P*_nisR_*). **(C)** Schematic drawing of NisK WT (left) and NisK mutant RKNlacZ-ΔKe (right). **(D)** Western-blotting assay of the membrane proteins extracted from *L. lactis* MG1363 RKNlacZ and *L. lactis* MG1363 RKNlacZ-ΔKe, respectively, probed by NisKc antiserum. **(E)** The β-galactosidase activities of strain *L. lactis* MG1363 RKNlacZ and *L. lactis* MG1363 RKNlacZ-ΔKe induced by gradient concentrations of nisin. MU stands for Miller Units which indicated the activity of β-galactosidase.

Many lantibiotics are sensed by HK named LanKs to regulate its own biosynthesis or other cellular processes. We analyzed the structures of known LanKs which sense specific lantibiotics by SMART database (Letunic et al., 2006). We found that there was a group of HK with two trans-membrane segments and an extracellular region (>100 amino acids) which is likely the sensor domain. **Figure 1A** showed NisK modular architecture which is similar to other LanKs in this group of HK. **Table 2** showed the examples of this type of HK including NisK (nisin as the inducer), SpaK (subtilin as the inducer), EtnK (entianin as the inducer), and so on. Since most peptide sensing HK are multi trans-membrane proteins and their sensing regions are associated with cell membrane, few report is focusing on the sensing mechanism of this type of rarely seen HK. Additionally, solute and nutrients are usually reported to be inducers of the first group of HK, how they sense peptide ligand is hardly known.

As NisK is well known to be induced by nisin, it is a good representative to study how this type of HK sense specific peptide pheromone. NisK protein contains 447 amino acids with predicted molecular mass of 51.5 kDa and shares several of the typical box-motifs (H, N, D, F, and G boxes) belonging to the HPK-3c subfamily HK (Grebe and Stock, 1999). The functional domain prediction performed by SMART database (Letunic et al., 2006) shows that NisK contains one extracellular region of 109 residues (from Leu36 to Asn144) and two *trans*-membrane domains (TMs) at the N-terminal, a His Kinase A phosphoacceptor (HisKA) domain and a HK like ATPase (HATPase_c) domain at the C-terminus (**Figure 1A**). The extracellular region in the N-terminal of NisK was predicted to sense nisin to initiate the signal transduction.

### The Extracellular Region of NisK was Involved in Nisin Recognition

In order to study, how NisK regulates nisin biosynthesis through the extracellular region, *nisRK* and a reporter gene *lacZ* under the control of promoter P*_nisA_* were cloned into vector pMG36e and transformed to *L. lactis* MG1363, forming a reporter strain *L. lactis* MG1363 RKNlacZ (**Figure 1B**). In this strain, *nisR* and *nisK* were constitutively expressed under the control of promoter P*_nisR_*. When NisK detects a certain concentration of nisin, it would be phosphorylated and activate NisR to initiate the expression of β-galactosidase which was under the control of P*_nisA._* Nisin biosynthesis regulated by NisK could be monitored by detecting the β-galactosidase activities in strain *L. lactis* MG1363 RKNlacZ.

In order to explore whether the extracellular region of NisK was involved in regulating nisin biosynthesis, most part of the extracellular region of NisK (from Ile39 to Ser140) in *L. lactis* MG1363 RKNlacZ strain was deleted (**Figure 1C**), forming a mutant strain *L. lactis* MG1363 RKNlacZ-ΔKe. The cytoplasmic region of NisK (NisKc) was expressed in *E. coli*, and purified to raise specific antibodies in mice. The anti-NisKc serum was used in the Western blotting assay to detect whether NisK mutant RKNlacZ-ΔKe could be located on the membrane. The corresponding RKNlacZ-ΔKe band (40 kDa) which was about 10 kDa less than full-length NisK (50 kDa) was detected in the membrane portion of cell lysate (**Figure 1D**), indicating that RKNlacZ-ΔKe was expressed in *L. lactis* MG1363 and anchored to the cell membrane. *L. lactis* MG1363 RKNlacZ exhibited an increased β-galactosidase activity after induced by increasing concentration of nisin, while the sensing ability of strain RKNlacZ-ΔKe was dramatically impaired and no obvious difference was detected when induced by raising concentration of nisin (**Figure 1E**). These results demonstrated that the extracellular region of NisK was necessary in regulating nisin biosynthesis.

### Important Conserved Residues Involved in Nisin Perception in NisK Extracellular Region

**FIGURE 2:**
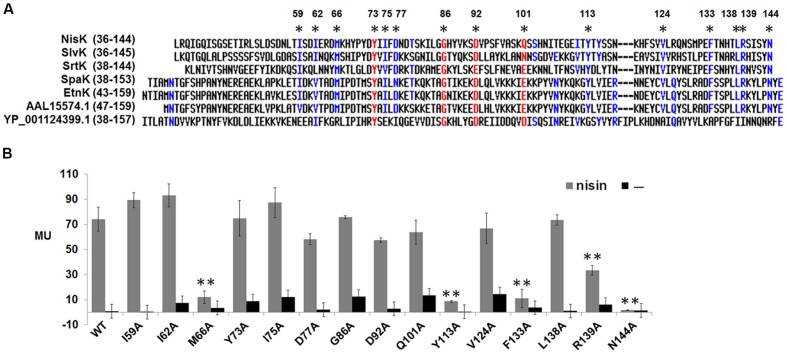
**Sequence alignment of extracellular regions of NisK-like HK and identification of key residues. (A)** Sequence alignment of NisK extracellular region with other extracellular domains from priedicted NisK-like HK SlvK, SrtK, SpaK, EtnK, HK AAL15574.1, and YP_001124399.1. Conserved residues were labled by **^∗^**. **(B)** β-galactosidase activities of *L. lactis* MG1363 RKNlacZ mutant strains containing NisK mutants in which conserved residues were substituted by alanine. 9 ng/ml nisin was used to induce these strains when needed. Strains induced by nisin were indicated by gray column and strains not induced were indicated by dark column. At least three independent experiments were performed in triplicate.

The full-length NisK was analyzed by online database PredictProtein^2^, but no known sensor domain was found in its extracellular region. As we shown in **Table 2** that a group of LanKs possessed two trans-membrane segments and a large extracellular region at the N-terminus, sequence alignments of their extracellular regions were carried out. The results showed that LanKs including NisK (YP_005867990.1), SlvK (AEX55158.1), SrtK (BAB08161.1), SpaK (AAB91593.1), EtnK (AEK64500.1), AAL15574.1, and YP_001124399.1, respectively, sensing nisin A, and nisin-like lantibiotics salivaricin D, streptin 1/2, subtilin, entianin, ericin S/A, geobacillin I harbored 15 conserved residues in their extracellular regions (**Figure 2A**).

**FIGURE 3:**
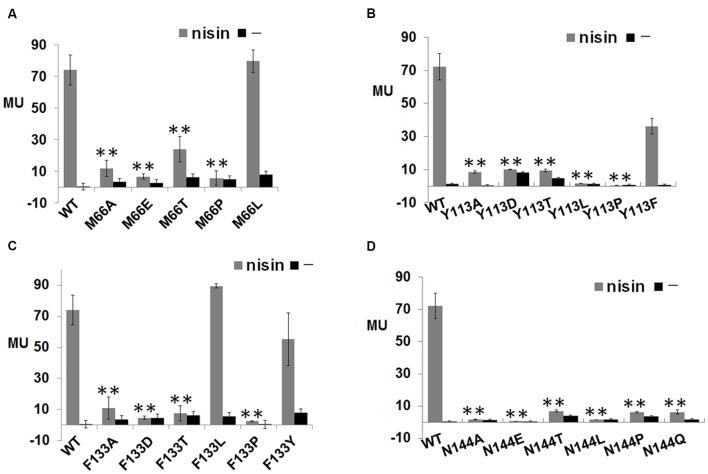
**β-galactosidase activities of *L. lactis* MG1363 RKNlacZ mutant strains. (A)** β-galactosidase activities of *L. lactis* MG1363 RKNlacZ mutant strains containing NisK mutants in which conserved Met 66 were substituted by several amino acids. **(B)** β-galactosidase activities of *L. lactis* MG1363 RKNlacZ mutant strains containing NisK mutants in which conserved Tyr 113 were substituted by several amino acids. **(C)** β-galactosidase activities of *L. lactis* MG1363 RKNlacZ mutant strains containing NisK mutants in which conserved Phe 133 were substituted by several amino acids. **(D)** β-galactosidase activities of *L. lactis* MG1363 RKNlacZ mutant strains containing NisK mutants in which conserved Asn 144 were substituted by several amino acids.

To test whether these 15 conserved residues were crucial for NisK to recognize nisin, each residue was substituted with alanine and the corresponding NisK mutants were constructed in the plasmid pMG36e-RKNlacZ by site-directed ligase-independent mutagenesis (SLIM) and *L. lactis* MG1363 RKNlacZ mutant strains were obtained after the transformations. The sensing abilities of NisK mutants were tested by monitoring the β-galactosidase activities of *L. lactis* MG1363 RKNlacZ mutant strains. The results showed that the β-galactosidase activities of strains with NisK mutants M66A, Y113A, F133A, and N144A were hardly detected, while others showed similar β-galactosidase activities compared with NisK WT (**Figure 2B**), demonstrating that these four residues were important for NisK to regulate nisin biosynthesis. When the hydrophobic Met66 was substituted by Leu which is also a hydrophobic amino acid, NisK maintained its regulation capacity. However, while other substitutions of Met66, such as negatively charged amino acid Asp, polar amino acid Thr or non-polar amino acid Pro greatly impaired the nisin biosynthesis regulation of NisK (**Figure 3A**). When the hydrophobic Phe133 was mutated into hydrophobic amino acids such as Leu and Tyr, NisK retained its regulation ability whereas other substitutions like Asp, Thr and Pro greatly impaired or eliminated the regulation ability of NisK (**Figure 3C**). These results demonstrated that the hydrophobic traits of the conserved residues Met66 and Phe133 were crucial for NisK to regulate nisin biosynthesis. When the hydrophobic and aromatic Tyr113 was substituted by aromatic amino acid Phe, nisin biosynthesis still remained about 50% compared to wild type, whereas other substitutions like Asp, Thr, Leu and Pro greatly impaired or eliminated nisin production (**Figure 3B**). This result demonstrated that the aromatic side chain of Tyr 113 were crucial for NisK to regulate nisin biosynthesis. As for the polar residue Asn144, mutation to any other amino acids almost eliminated nisin production (**Figure 3D**).

### Important Hydrophobic Region in NisK Extracellular Sensor Domain

**FIGURE 4:**
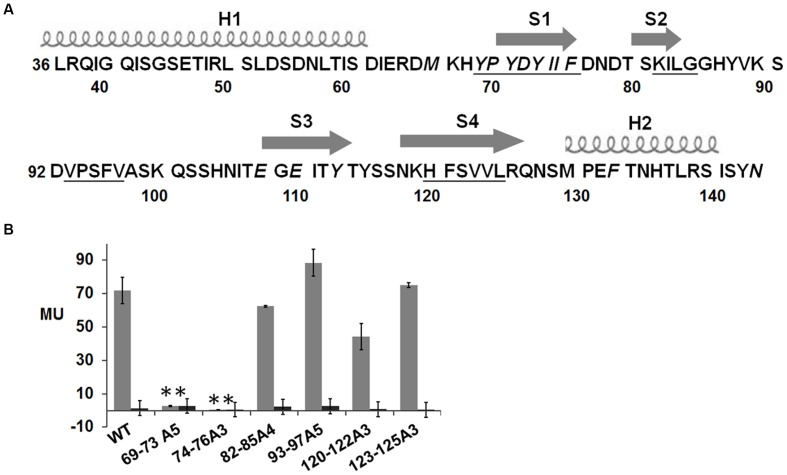
**Schematic drawing of secondary structure of NisK extracellular region and β-galactosidase activities of *L. lactis* MG1363 RKNlacZ mutant strains. (A)** Schematic drawing of the predicted secondary structure of NisK extracellular region. Two helixes (H1 and H2) were labeled by screws and four strands (S1, S2, S3, and S4) were labeled by arrows. The calculated four hydrophobic zones were labeled by underlines. **(B)** β-galactosidase activities of *L. lactis* MG1363 RKNlacZ strains containing NisK mutants in which the tandem hydrophobic residues in each hydrophobic region were all substituted by alanine. Strains induced by 9 ng/ml nisin were indicated by gray column and strains not induced were indicated by dark column. ^∗^Values and standard deviation are calculated from at least three independent experiments performed in triplicate. Significance (*p* < 0.01) was determined by Student’s *t*-test.

The secondary structural analyses by PredictProtein showed that there were two helices and four strands in the extracellular region of NisK (**Figure 4A**). The hydrophobicity or hydrophilicity scales of NisK extracellular region were also calculated by ExPASy ProtScale^3^. Four zones rich in hydrophobic amino acids including residues YPYDYIIF(69–76), KILG(82–85), VPSFV(93–97), and HFSVVL(120-125) were found (**Figure 4A**). To detect whether these hydrophobic zones were involved in regulating nisin biosynthesis, NisK mutants 69–73A5, 74–76A3, 82–85A4, 93–97A5, 120–122A3, and 123–125A3, in which tandem residues in each hydrophobic zone were all substituted by alanines, were constructed in *L. lactis* MG1363 RKNlacZ, and their β-galactosidase activities were tested. The mutants 69–73A5 and 74–76A3 which were located in the first strand showed greatly reduced or no β-galactosidase activities, while other mutants still maintained similar regulation abilities to NisK (**Figure 4B**). These results indicated that the first strand which was rich of hydrophobic amino acids in NisK extracellular region was necessary to regulate nisin biosynthesis.

### Important Charge Residues in NisK Extracellular Sensor Domain

**FIGURE 5:**
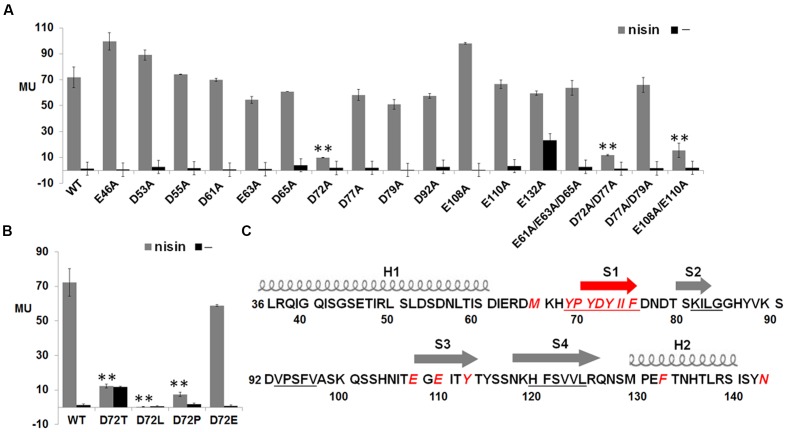
**β-galactosidase activities of *L. lactis* MG1363 RKNlacZ strains containing NisK mutants with anionic residues mutated and key residues in NisK for sensing nisin. (A)** β-galactosidase activities of *L. lactis* MG1363 RKNlacZ strains containing NisK in which anionic residues were substituted by alanine. **(B)** β-galactosidase activities of *L. lactis* MG1363 RKNlacZ mutant strains containing NisK mutants in which the important Asp 144 were substituted by several amino acids. Strains induced by 9 ng/ml nisin were indicated by gray column and strains not induced were indicated by dark column. ^∗^Values and standard deviation are calculated from at least three independent experiments performed in triplicate. Significance (*p* < 0.01) was determined by Student’s *t*-test. **(C)** Schematic drawing of the predicted secondary structure of NisK extracellular region and the crucial residues for NisK sensing nisin. Two helixes (H1 and H2) were labeled by screws and four strands (S1, S2, S3, and S4) were labeled by arrows. Most crucial residues were concentrated in or near S1. The calculated four hydrophobic zones were labeled by underlines and the crucial residues were displayed by red letters.

The electrostatic interaction was a kind of common force in protein–protein interaction, and has been reported to play an important role in the recognition between BovK and bovicin HJ50 (Teng et al., 2014). To discover whether anionic residues in NisK extracellular region were necessary to identify cationic peptide nisin and affect nisin biosynthesis, each anionic residue in NisK extracellular region was replaced with alanine based on the plasmid pMG36e-RKNlacZ. The mutated plasmids were transformed into *L. lactis* MG1363 to produce mutant strains E46A, D53A, D55A, D61A, E63A, D65A, D72A, D77A, D79A, D92A, E108A, E110A, and E132A. The β-galactosidase activity of the mutant strain D72A was severely decreased, while other mutant strains did not show obvious differences compared with WT (**Figure 5A**). These results indicated that the Asp72 was vital for NisK to regulate nisin biosynthesis. To determine whether the negative charges of Asp72 was necessary, the aspartic acid was replaced by Thr, Leu, Pro, and Glu respectively. The β-galactosidase activity assay of each mutant was performed, and the results indicated that the mutant strain with NisK D72E remained similar regulation capacity with WT, while mutants with other substitutions showed significantly decreased regulation capacities (**Figure 5B**). This indicated that the negative charge of aspartic acid residue at position 72 was crucial for NisK to regulate nisin biosynthesis. Four NisK mutants with more than one anionic residues changed were further created, yielding mutant strains *L. lactis* MG1363 RKNlacZ D61A/E63A/D65A, D72A/D77A, D77A/D79A, and E108A/E110A. The β-galactosidase activities of mutants D61A/E63A/D65A and D77A/D79A did not display obvious difference with wild type, while D72A/D77A and E108A/E110A demonstrated remarkably decreased regulation abilities (**Figure 5A**). Nisin biosynthesis regulate by mutant E108A/E110A was decreased, while single mutant E108A and E110A did not, demonstrating that negative charges in Glu108 and Glu110 might work synergistically to influence nisin biosynthesis. **Figure 5C** showed a summary of residues that were proved to be important for NisK to regulate nisin biosynthesis.

### Nisin Binding Abilities of NisK and Key Mutants

**FIGURE 6:**
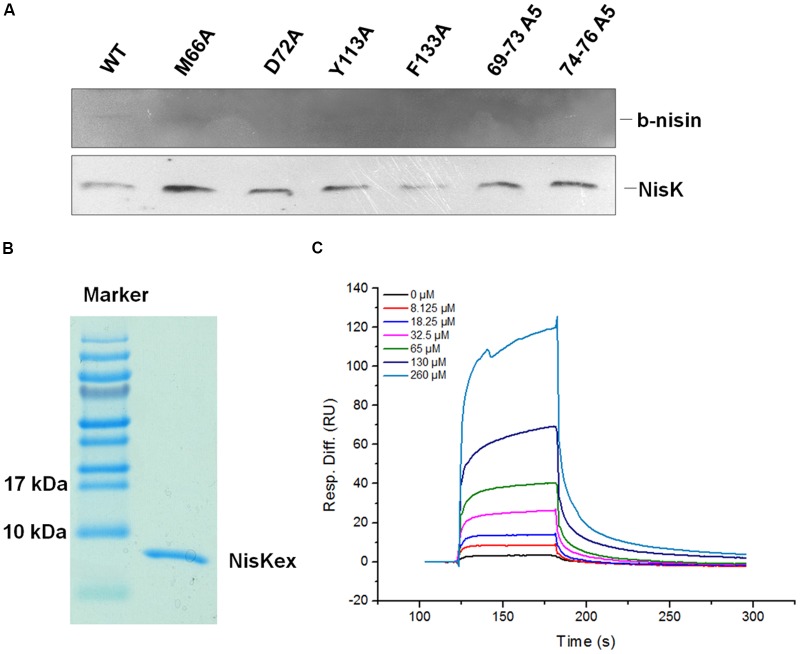
**Analysis of NisK mutants interacting with nisin. (A)** Far-Western blot and Western blot analysis of b-nisin binding to NisK. Membrane proteins from NisK and NisK mutants strains were probed with b-nisin (up) and NisK antiserum (down). **(B)** NisK extracellular region (NisKex) was purified and isolated in SDS-PAGE gel. **(C)** SPR analyses of interaction between NisKex and nisin. NisKex was bond to the chip as ligand and the analyte nisin was tested at gradient concentrations as indicated, *K*_D_ = 5.38e^-4^ M.

In order to clarify whether the important residues in NisK affected the interaction between nisin and NisK, the membrane proteins from *L. lactis* MG1363 RKNlacZ strain and the mutant strain M66A, D72A, Y113A, F133A, 69–73A5, and 74–76A3 were extracted. Nisin was labeled by biotin, and the modified product was named by b-nisin. The interaction between b-nisin and membrane proteins was studied by far-Western blotting assay. As **Figure 6A** shown that incubation of the membrane proteins with b-nisin labeled a membrane protein of about 51.5 kDa, corresponding to NisK (51.5 kDa) binding to b-nisin. Western blotting analysis utilizing polyclonal NisKc antiserum against the same proteins showed that NisK was visualized at the position of about 51.5 kDa. This result indicated that nisin could interact with NisK, although the binding affinity is weak. The membrane protein of strain M66A, D72A, Y113A, F133A, 69–73A5, and 74–76A3 did not show b-nisin binding bands. These results indicated that these key residues in the first strand of NisK extracellular region and Phe 133 were important for NisK to identify nisin and regulate nisin biosynthesis.

Additionally, *in vitro* interaction between nisin and NisK extracellular region (NisKex) was also detected by SPR. The extracellular region of NisK tagged by His_6_ was expressed in *E. coli* and purified (**Figure 6B**). The interaction between NisKex and nisin was performed by SPR. His_6_-NisKex was immobilized on a CM5 chip, and nisin was injected through this chip as an analyte. The concentrations of nisin were in twofold gradient dilution from 260 to 8.125 μM while 0 μM nisin was also injected as a control. The SPR curves were fitted by a 1:1 (Langmuir) binding model and local fitted *R*max to get the equilibrium constants *K_D_*, which was regarding to the protein–protein affinity. The curves indicating the binding abilities of different concentration of nisin to NisKex were shown in **Figure 6C**. The association rate (ka) of NisKex and nisin was 65.21/Ms, dissociation rate (kd) was 1/s, and the *K_D_* value was about 5.38e^-4^M, indicating that the binding force between nisin extracellular region and NisKex was comparable weak. We supposed that NisKex may not fold properly in the absence of the trans-membrane segments.

## Discussion

Two-component regulatory systems containing HK and RR are crucial for bacterial to sense the stimuli and transduce the signals. Most of the reported HK that sense peptide signals are multi-trans-membrane proteins, and their N-terminal sensing regions were predicted to be associated with cell membrane (Mascher et al., 2006). By *in silico* analysis we found a rarely reported group of HK with two trans-membrane domains and one extracellular region which sense lantibiotics as ligands. We revealed the key regions and residues in the representative NisK sensor domain for recognizing nisin, and our work might revealed some principles about how Group I HK recognize peptide pheromones.

Deletion of the extracellular region of NisK did not affect its localization on cell membrane, but destroyed nisin biosynthesis, demonstrating that the extracellular region was important for NisK to regulate nisin biosynthesis. There are two reasons that nisin biosynthesis was impaired. One is that the extracellular region was responsible for recognizing nisin, and the other is that the deletion of extracellular region affected the structure of NisK, and resulted in the impair of signal transduction through NisK to NisR and downstream gene expression. We are not sure whether the destroyed nisin production in NisK mutant strain was caused by the loss of sensing ability or signal transduction capacity. However, there is no doubt that nisin biosynthesis was impaired by the deletion of NisK extracellular region which is predicted to be the sensor region of NisK. Most of known Group I HK recognize their signal molecules through the extracellular region, and several specific sensing domains, such as PDC domain (mixed alpha-beta folds) or NarX-like sensor domain (all alpha folds) were identified through mutagenesis or crystal structure analysis. However, we know little about the function of NisK sensor and did not find known sensing domains in NisK extracellular region. We have attempted to analyze the crystal structure of NisK but failed because of the poor quality of NisK protein crystal.

Mutagenesis analysis in NisK extracellular region were applied to minimize the effect of structure changes of NisK and find out the key sites in NisK for regulating nisin biosynthesis. In other LanKs including SlvK, SrtK, SpaK, EtnK and so on belonging to this type of HK, no reported sensing domain was found too. Sequence alignment revealed some conserved residues in their extracellular regions, and our results showed that four conserved residues Met66, Tyr113, Phe133, and Asn144 in NisK are involved in regulating nisin biosynthesis. Further substitutions of each important residues with other amino acids demonstrated that the hydrophobicity of Met66 and Phe133, the aromatic side chain of Tyr113 are involved in regulating nisin biosynthesis. We supposed that these residues in other NisK-like HK might also contributed to regulate their corresponding lantibiotics biosynthesis. As for the polar residue Asn144, each substitution with non-polar amino acids Ala, Leu, Pro, polar amino acids Thr, Gln, and negatively charged Glu almost eliminated the regulation ability of NisK. This residue reside in the end of the extracellular region (**Figure 5C**) and very near the *trans*-membrane domain of NisK, as a result, we supposed that Asn144 might function in signal transduction from which needs further research to verify.

The predicted secondary structure of NisK extracellular region exhibited four short β-strands flanked by two α-helixes on the ends. By testing the regulation abilities of different NisK mutants, some regions and sites in NisK extracellular region were found to be the key sites. Hydrophobic zones YPYDY (69–73) and IIF (74–76), negatively charged residue Asp72 were all located in the first predicted strand (S1). Thus we proposed that the strand S1 might be the most important region in NisK extracellular region that was involved in regulating nisin biosynthesis. Besides, two conserved aromatic residue Tyr113 and Phe133 were also very important for NisK to interact with nisin. Because we failed to obtain NisK protein crystal with high quality, we did not know the crystal structure of NisK and how S1 would work with Tyr113 and Phe133 needs further research.

Our far-Western blotting assay indicated that nisin could interact with NisK directly. It is reported that nisin bind to cell membrane through electrostatic interactions with phospholipid head groups in the membrane because of its cationic nature (Bauer and Dicks, 2005). It is also suggested that nisin is adsorbed to the membrane surface for acting as a quorum sensing signal (Ra et al., 1996; Hilmi et al., 2006), which could explain why only weak binding force was detected between nisin and NisK extracellular region by SPR. As **Figure 6C** shown that nisin dissociated from NisK very fast, we supposed that nisin-NisK interaction is not stable, and nisin might perform a “touch and go away” model to interact with NisK. Besides, we supposed that NisKex may not fold properly in the absence of the trans-membrane segments which might also be another reason that nisin was detected to have weak affinity to the extracelluar region of NisK protein. Thus we supposed that NisK recognized nisin might also be related to the cell membrane which needs further study to be verified.

Gram-positive bacteria recognize diverse post-translationally modified peptides using either trans-membrane HK or cytoplasmic receptors. The discovering of countless new peptide signaling systems in the Gram-positive bacteria highlights our current limited understanding of this emerging type of quorum-sensing signal molecules. And this will lead to further structure-based investigations for better understanding of specific peptide-receptor interactions.

## Author Contributions

JZo and KT designed research. XG, KT, JW, FZ, and JZa contributed to experimental work. XG and KT finished the data analysis and wrote the manuscript. KT and JZo revised the manuscript.

## Conflict of Interest Statement

The authors declare that the research was conducted in the absence of any commercial or financial relationships that could be construed as a potential conflict of interest.

## Footnotes

1 http://smart.embl-heidelberg.de/

2 http://ppopen.informatik.tu-muenchen.de/

3 http://web.expasy.org/protscale/
